# Does plant immunity play a critical role during initiation of the legume-rhizobium symbiosis?

**DOI:** 10.3389/fpls.2015.00401

**Published:** 2015-06-02

**Authors:** Katalin Tóth, Gary Stacey

**Affiliations:** Division of Plant Sciences and Biochemistry, Christopher S. Bond Life Sciences Center, National Center for Soybean Biotechnology, University of Missouri-ColumbiaColumbia, MO, USA

**Keywords:** legume, root nodule symbiosis, plant immunity, receptor-like kinase, nod factor, lipo-polysaccharides

## Abstract

Plants are exposed to many different microbes in their habitats. These microbes may be benign or pathogenic, but in some cases they are beneficial for the host. The rhizosphere provides an especially rich palette for colonization by beneficial (associative and symbiotic) microorganisms, which raises the question as to how roots can distinguish such ‘friends’ from possible ‘foes’ (i.e., pathogens). Plants possess an innate immune system that can recognize pathogens, through an arsenal of protein receptors, including receptor-like kinases (RLKs) and receptor-like proteins (RLPs) located at the plasma membrane. In addition, the plant host has intracellular receptors (so called NBS-LRR proteins or R proteins) that directly or indirectly recognize molecules released by microbes into the plant cell. A successful cooperation between legume plants and rhizobia leads to beneficial symbiotic interaction. The key rhizobial, symbiotic signaling molecules [lipo-chitooligosaccharide Nod factors (NF)] are perceived by the host legume plant using lysin motif-domain containing RLKs. Perception of the symbiotic NFs trigger signaling cascades leading to bacterial infection and accommodation of the symbiont in a newly formed root organ, the nodule, resulting in a nitrogen-fixing root nodule symbiosis. The net result of this symbiosis is the intracellular colonization of the plant with thousands of bacteria; a process that seems to occur in spite of the immune ability of plants to prevent pathogen infection. In this review, we discuss the potential of the invading rhizobial symbiont to actively avoid this innate immune response, as well as specific examples of where the plant immune response may modulate rhizobial infection and host range.

## Introduction

The root nodule symbiosis (RNS) is one of the most fascinating, yet not completely understood beneficial host–microbe interactions. RNS is limited to the FaFaCuRo (Fabales, Fagales, Cucurbitales, and Rosales) clade that belongs to Eurosid I plants (Kistner and Parniske, 2002). Under nitrogen limiting conditions, many legume plants are infected by nitrogen-fixing soil bacteria, termed rhizobia. Subsequent to an initial signal exchange between host and symbiont, the bacteria enter the host root usually through epidermal root hair cells. An infection thread (IT) of plant origin is formed that extends and eventually delivers the rhizobia into newly dividing cortical cells. These cells give rise to a nodule primordium that develops into the nodule, a new root organ. In the nodule, bacteria differentiate into bacteroids, the nitrogen-fixing form of rhizobia, which reduces atmospheric dinitrogen into ammonia that is used by the host plant. In exchange, the bacteria receive a steady carbon source provided by plant photosynthesis.

Although the first observation of legume nodulation was reported a few 100 years ago, we still do not fully understand the underlying mechanisms that maintain a perfect balance between host and symbiont to allow such an intimate symbiosis to develop. Among the exciting new findings is a growing recognition that the plant immune system is active during RNS. In this review, we will point out recent observations to indicate when and how the host plant immune system acts to control nodule formation and host range.

### Rhizobia are Part of a Diverse and Active Rhizosphere Microbiota

In the soil, there is an extremely large population of microorganisms that keep the soil ecosystem functioning. For instance, a metagenomics study of the *Arabidopsis thaliana* rhizosphere revealed 43 bacterial phyla and divisions (Bulgarelli et al., 2012). Microorganisms of the rhizosphere (part of the soil directly surrounding and impacted by the root) interact with the roots, providing nutrients and protection against biotic and abiotic stress. Specific rhizosphere microbes also have the ability to enter the root and become inter- or intracellular inhabitants, sometimes contributing to plant growth and development (Bulgarelli et al., 2012; Lundberg et al., 2012). Given the diversity of rhizosphere microbes and the potential threat for the plant, it is not surprising that plants have the ability to distinguish threatening intruders (i.e., pathogens) from beneficial microbiota.

Hundreds of different microorganisms are attached to the surface of a root. Leguminous plants under nitrogen limiting conditions secrete secondary metabolites (e.g., flavonoids) that can signal to and recruit compatible, symbiotic rhizobia (Oldroyd et al., 2011). Specific flavonoids act as inducers of the rhizobial nodulation genes, which encode the enzymes needed for synthesis of the lipo-chitooligosaccharide (LCO) nodulation factor [Nod factors (NF)], the key rhizobial signaling molecule that elicits the first plant responses in establishing RNS (Fisher and Long, 1992).

### Parallels between Symbiont- and Pathogen-Triggered Responses

The term ‘microbe-associated molecular pattern’ (MAMP) is used for specific recognition signatures found in conserved molecules [e.g., bacterial flagellin, cell wall components like lipopolysaccharide, chitin and peptidoglycan (PGN)] derived from microbes, usually pathogens that infect both plants and animals (Ausubel, 2005). MAMPs are characterized by their ability to induce an innate immune response in the host. Therefore, NF is usually not considered a MAMP since it induces nodule formation on the host, as opposed to inducing immunity. However, NF can induce some responses that are normally associated with plant innate immunity (Day et al., 2001; Ramu et al., 2002; Pauly et al., 2006). This is perhaps not surprising since longer chain chitin oligomers (degree of polymerization > 6) are strong inducers of plant innate immunity (Liang et al., 2014). Unlike simple chitin, NF is a LCO molecule comprised of an *N*-acetylglucosamine backbone with site-specific decorations and an *N*-acyl chain (D’Haeze and Holsters, 2002). The addition of very low concentrations of NF (<10 nM) was shown to induce a variety of responses on the compatible legume hosts. These include plasma membrane depolarization, perinuclear calcium spiking, cytoskeletal changes, root hair deformation, induction and repression of gene expression and, in a few plant species, induction of nodule primordia (D’Haeze and Holsters, 2002; Oldroyd and Downie, 2008).

Responses elicited by MAMP perception have been well-studied in many plants (De Coninck et al., 2015). These include generation of reactive oxygen species (ROS), cytosolic Ca^2+^ elevations, activation of mitogen-activated protein kinase (MAPK) and calcium-dependent kinases, callose deposition and defense-related gene expression (Boller and Felix, 2009; Greeff et al., 2012). However, in comparison with leaves, less attention has been paid to MAMP responses in roots even though many pathogens do invade via roots. MAMP-triggered immune signaling in *Arabidopsis* roots occurs in a similar fashion to leaves (Millet et al., 2010; De Coninck et al., 2015). Roots of seedlings responded by callose deposition to MAMPs like flg22 (a peptide molecule originating from bacterial flagellin), PGN and chitin. Callose deposition was observed in the root elongation zone in response to flg22 and PGN, while chitin elicited callose deposition in the root maturation zone (Millet et al., 2010), indicating the ability of different root tissues to distinguish between these MAMPs (De Coninck et al., 2015).

### Receptor-Like Kinases Involved in Symbiotic and/or Immune Signaling

Microbe-associated molecular patterns are recognized by pattern recognition receptors (PRRs) localized at the cell surface, including receptor-like kinases (RLK) and receptor-like proteins (Zipfel, 2014). The extracellular region of RLKs can be composed of lysin motif (LysM)-domains (LysM-RLK) and/or leucine-rich repeats (LRR-RLK), both of which are involved in microbe detection (Greeff et al., 2012).

NF is perceived by RLKs with an extracellular domain of 2–3 LysM domains, a single membrane-spanning region and an active or inactive intracellular kinase domain. These LysM-RLKs were identified in model legume species such as LjNFR1/LjNFR5 (NF Receptor 1 and 5) in *Lotus japonicus*, GmNFR1/GmNFR5 in soybean (*Glycine max*), and LYK3/NFP [Lysin motif receptor-like kinase 3 and NF Perception (NFP)] in *Medicago truncatula* (Amor et al., 2003; Limpens et al., 2003; Madsen et al., 2003; Radutoiu et al., 2003; Indrasumunar et al., 2010, 2011). Mutations in these genes significantly alter nodulation capability of the legume host.

The data suggest that the NF receptor is composed of a heterodimer or, perhaps, heterotetramer. LjNFR5 binds NF with higher affinity than LjNFR1 (Broghammer et al., 2012). However, LjNFR5 or MtNFP lack kinase activity (Arrighi et al., 2006; Madsen et al., 2011) and, therefore, likely signal by activation of the NFR1 or LYK3, respectively, kinase domain. Co-expression of LjNFR1 and LjNFR5, as well as MtNFP and MtLYK3, in a heterologous *in planta* tobacco system induced strong defense responses in the absence of NF (Madsen et al., 2011; Pietraszewska-Bogiel et al., 2013). These responses were similar to those elicited by over-expression of CERK1 in *Arabidopsis*, another LysM-RLK. AtCERK1 has an active intracellular kinase domain and functions as a heterotetramer with AtLYK5, which lacks a functional kinase domain, to recognize long-chain chitooligosaccharides (dp > 6) to induce plant immune responses (Cao et al., 2014; Liang et al., 2014).

Recently, it was shown that the rice MAMP receptor OsCERK1, is also required for establishment of symbiosis with mycorrhizal fungi (Miyata et al., 2014; Zhang et al., 2015). Similar to rhizobia, establishment of this symbiosis also involves a LCO signal, called Myc factor, as well as short-chain chitooligosaccharides (dp < 6; Maillet et al., 2011; Genre et al., 2013). OsCERK1 displays the highest homology with LjNFR1. Therefore, a possible role of LjNFR1 and MtLYK3 in mycorrhization was tested with the results implicating both in the establishment of this symbiosis (Zhang et al., 2015). In *M. truncatula*, MtNFP was shown to be involved in the response to root oomycete pathogen *Aphanomyces euteiches, nfp* mutant plants were more susceptible to the oomycete than wild type plants (Rey et al., 2013). Indeed, recently, mutations in a number of *M. truncatula* symbiotic genes were shown to affect the ability of *Phytophtora palmivora* to infect roots; again emphasizing the overlap between symbiont and pathogen response (Rey et al., 2015).

Taken together, the data support the hypothesis that chitin and LCO reception are functionally related with the latter likely evolving from the more wide-spread and ancient chitin recognition system (Liang et al., 2014). The fact that, in some species, CERK1 and its orthologs function both in pathogen and symbiont recognition argue that this step may not be involved in discerning the beneficial or detrimental nature of the infecting microbe. This is a rather heretical view given the dogma from earlier studies that argued that LCO reception plays a key role in host range determination (Oldroyd et al., 2011).

### Do Rhizobia Suppress the Plant Immune System?

**FIGURE 1:**
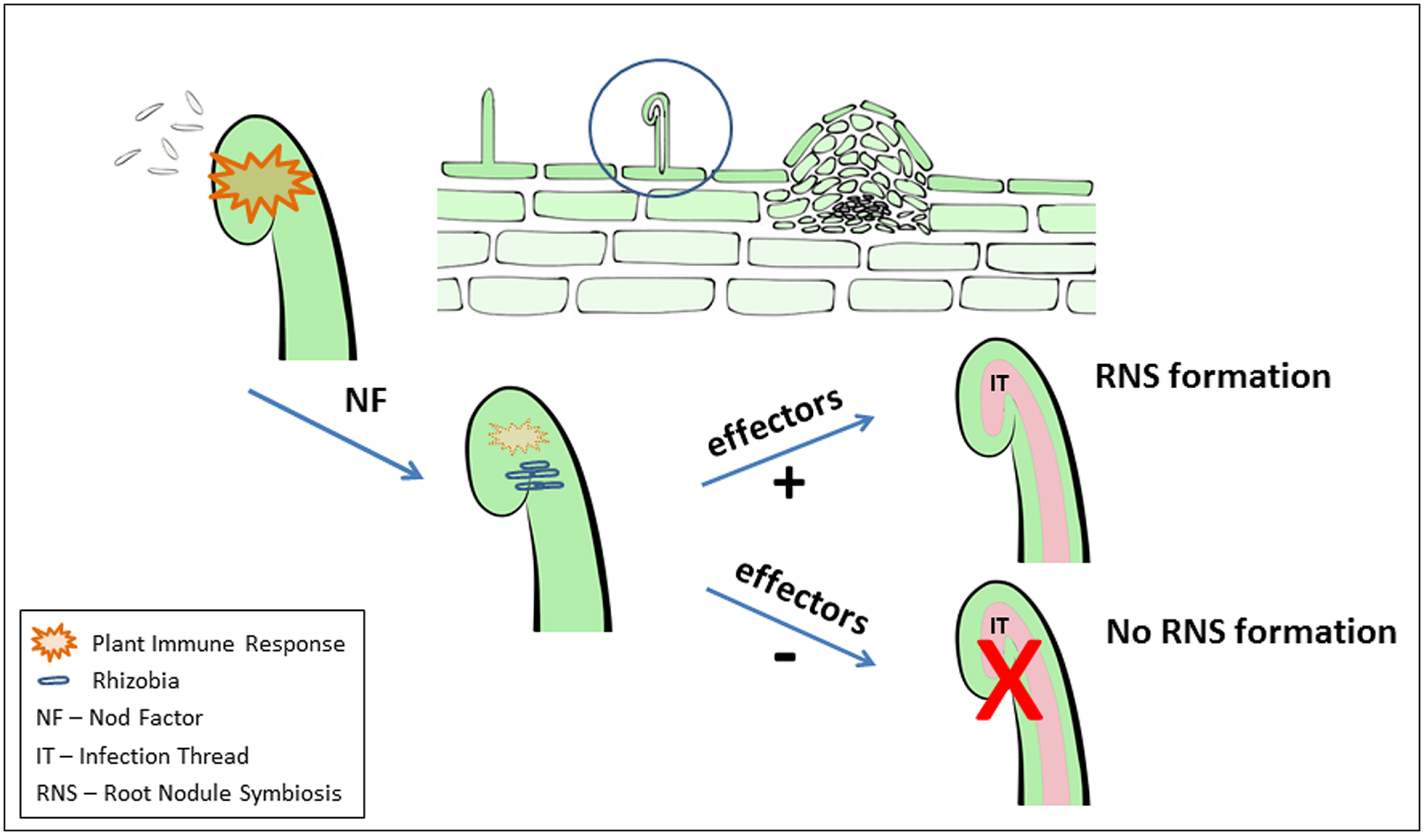
**Schematic illustration of elicitation and suppression of immune responses by Rhizobia during root nodule symbiosis (RNS) formation**. As discussed in the manuscript, during the early events of initiation of the legume-rhizobia symbiosis, a plant immune response is induced (illustrated by orange). In addition to acting as a signal to induce RNS development the Nod factor signal also acts to suppress this immune response. Bacterial effector proteins (e.g., delivered through a T3SS) can act to either negatively or positively modulate RNS. Strictly for the purposes of illustration, these events are shown as acting on a single root hair. However, clearly the situation is much more complex and it is likely that the plant immune response can impact RNS at various steps during its development.

The question whether the plant immune system might be involved in RNS is an obvious one considering the intimacy of the RNS (Fisher and Long, 1992). Unfortunately, this question has not received a great deal of direct, experimental examination. However, there are a number of observations that are consistent with a rapid, defense-like response occurring in legumes when infected by rhizobia (**Figure 1**). For instance, strong production of ROS was observed on alfalfa roots in response to the compatible symbiont *Sinorhizobium meliloti* (Santos et al., 2001). Transient and rapidly elevated ROS levels were observed on common bean *Phaseolus vulgaris* root hairs upon NF addition at physiological concentration (10^-9^M; Cardenas et al., 2008). Silencing of NADPH oxidase, required for ROS production, resulted in aborted IT formation and reduced nodule numbers on common bean roots (Montiel et al., 2012). The results suggest that ROS production is necessary for infection initiation but prolonged, elevated levels could be detrimental to nodulation.

A hypersensitive, cell death response was also reported on alfalfa roots in response to *S. meliloti* (Vasse et al., 1993). These results are consistent with recent large-scale transcriptomic and phosphoproteomic studies, performed on soybean and *M. truncatula* in response to their symbiotic rhizobia or purified NF, that revealed rapid induction of defense-related gene expression, as well as phosphorylation of proteins known to be involved in plant immune responses (Libault et al., 2010; Nguyen et al., 2012; Rose et al., 2012).

The levels of salicylic acid (SA), a key secondary signal involved in plant innate immunity (An and Mou, 2011), were found to increase in alfalfa roots upon inoculation with NF-defective (*nodC* mutant) rhizobia (Martínez-Abarca et al., 1998). Indeed, transgenic roots in which SA levels were reduced by expression of NahG, showed increased rhizobial infection (Stacey et al., 2006). Similarly, a number of other phytohormones, also involved in plant innate immunity, can affect the RNS (e.g., jasmonic acid; Ding and Oldroyd, 2009).

If the plant does mount a defense response to invading rhizobia, then, by analogy to bacterial pathogens, it is possible that rhizobia also have the ability to actively suppress this response. Indeed, suppression of immune responses, such as ROS production and SA accumulation, was demonstrated in *M. truncatula* and *M. sativa* roots upon addition of NF (Martínez-Abarca et al., 1998; Shaw and Long, 2003). In addition, down-regulation of a *PR2* (pathogenesis-related protein) homolog in *M. truncatula* was reported in response to *S. meliloti* inoculation, while a *S. meliloti* mutant defective in NF synthesis failed to induce the same response (Mitra and Long, 2004). Surprisingly, NF application can suppress defense responses not only in legumes but also non-legumes, such as *Arabidopsis*, tomato, and corn. For example, *Arabidopsis* leaves pre-treated with flg22 elicit a strong innate immune response that was suppressed by addition of NF (Liang et al., 2013). These findings suggest that LCO/NF might have a dual role in actively inducing RNS development while also actively suppressing plant immunity, which could inhibit RNS (**Figure 1**).

### Nodulation without Nod Factor Signaling Reveals a Key Role for Plant Innate Immunity in RNS

The dogma that existed for many years in the field of RNS research is that nodulation cannot occur in the absence of NF signaling. Thus, it was quite surprising when some rhizobia were found to nodulate specific *Aeschynomene* species in the complete absence of the nodulation genes, required for NF synthesis (Giraud et al., 2007). More recently, Okazaki et al. (2013) showed that a nodulation defective, *nfr1* mutant of the soybean cultivar Enrei could be nodulated by a *Bradyrhizobium elkanii* mutant unable to produce NF. Even more surprising was the finding that nodulation by this mutant was dependent on an active type III secretion system (T3SS). Microarray analysis revealed that symbiosis marker genes such as *ENOD40* and *NIN* were induced in the *nfr1* mutant suggesting T3SS-induced signaling (Okazaki et al., 2013). In plant pathogens, the T3SS secretes effector proteins directly into the plant cell that can enhance infection or, when the appropriate R protein is present, induce effector-triggered immunity (ETI; Boller and Felix, 2009).

Effectors are directly or indirectly perceived by nucleotide-binding site-LRR (NBS-LRR) receptors encoded by R (resistance)-genes (Boller and Felix, 2009). In soybean, *Rj2* and *Rfg1* alleles were found to restrict nodulation in a strain-specific manner; that is, while *Rj2* prevents nodulation with certain *B. japonicum* strains, *Rfg1* restricts the symbiosis with at least one *S. fredii* strain (i.e., USDA257; Yang et al., 2010). Tsukui et al. (2013) showed that the incompatibility of *B. japonicum* (USDA122) with *Rj2* soybean genotypes is mediated by the T3SS. This type of strain-specificity seems very analogous to the race-specificity of plant pathogens that is known to be determined by ETI. Kimbrel et al. (2013) examined Type III effector genes in *S. fredii* and *B. japonicum* and found that these genes exhibit a high degree of conservation in comparison to those secreted by pathogens.

The results of Okazaki et al. (2013) stand out since, for the first time, they suggest that the T3SS and associated effector proteins play a central role in RNS establishment. However, it remains to be determined which of the various *B. elkanii* effectors are required for nodule formation on soybean cv. Enrei. There is a wealth of earlier literature that supports a role for rhizobial effectors in modulating host range. Much of this work was done using *Rhizobium sp*. NGR234, which exhibits a very extended host range providing a variety of host species on which to examine nodulation (Perret et al., 2000). For example, the effector NopL from *Rhizobium* sp NGR234, when expressed in tobacco and *L. japonicus* was shown to suppress pathogen induction of PR protein expression and to interfere with MAPK signaling (Bartsev et al., 2004; Zhang et al., 2011). The dominant *Rj4* allele in soybean encodes a PR protein that was found to restrict soybean nodulation with certain *B. elkanii* and *B. japonicum* strains. These strains were restricted in infection of the epidermal cell layers of wild soybean (*G. soja*) roots (Hayashi et al., 2014; Tang et al., 2014). Perhaps relevant to the work on soybean, the *S. fredii* effector NopP and the *B. japonicum* effectors NopE1 and NopE2 were shown to be directly transported into the host plant cells of *Vigna* roots (Schechter et al., 2010; Wenzel et al., 2010). Both NopE and NopT exhibit protease activity. *B. japonicum* effector NopT1 triggered cell death response when expressed in tobacco, while the NopT2 did not induce the same response (Dai et al., 2008; Kambara et al., 2009; Fotiadis et al., 2012). As mentioned earlier, strong ROS production was observed in response to NF application (Cardenas et al., 2008). NGR234 NopM (an E3 ubiquitin ligase) effector expressed in tobacco inhibited ROS production, while inducing defense-related gene expression (Xin et al., 2012).

Published data suggest that the need for an active effector secretion system (e.g., T3SS) is widespread in legumes. For example, wild type *M. loti* (MAFF303099) is not able to infect *Leucaena leucocephala* (a mimosoid tree), while the T3SS mutant was able to efficiently nodulate this same species (Hubber et al., 2004; Sánchez et al., 2009). Not all rhizobia possess a T3SS but in these cases other systems may operate. For example, *M. loti* strain R7A, *S. meliloti* and *R. etli* possess a type IV secretion system (T4SS; Soto et al., 2006). Deletion of T4SS in *M. loti* strain R7A extended the nodulation host range to include *L. leucocephala*, which is not nodulated by the wild type strain (Hubber et al., 2004). On the other hand, mutation of the T4SS in *S. meliloti* did not seem to impact formation of a functional symbiosis on alfalfa roots (Jones et al., 2007).

### Conclusion and Future Perspectives

Some 29 years ago, our laboratory published a review that sought to compare and contrast rhizobium, agrobacterium and pathogen infection of plants (Halverson and Stacey, 1986). Therefore, it is satisfying to now see how many interesting parallels have been documented between rhizobial–plant, mycorrhizal–plant, and pathogen–plant interactions. For example, MAMP signaling and the associated receptors are clearly relevant to these associations. It is now well accepted that LCO and chitin signaling share similar receptors, reflecting an evolutionary connection. Indeed, in some cases, the chitin receptor plays a dual role in recognizing plant fungal pathogens, while also promoting symbiotic development.

When well established dogma in any field gets overturned, it means that research progress is being made. An example in the rhizobial field is the realization that nodulation does not *sensu stricto* require NF production. In the case of soybean, nodulation can occur without NF but this requires an active T3SS. Although unidentified, the assumption is that rhizobial effector proteins are exported to the soybean host that is allowing nodulation to occur (**Figure 1**). The parallels to plant–pathogen interactions are clear, where effectors can either promote virulence or resistance. R proteins are clearly important in the rhizobial symbiosis, at least in modulating host range. At this point, the role of effectors and R proteins in RNS cannot be refuted. However, perhaps the more interesting question is whether these components are necessary, perhaps essential, for nodule formation either mediated by NF or not. The case in soybean cv. Enrei clearly argues for an essential role but could the research focus on NF signaling be hiding a general, essential role in RNS in other plant species?

Regardless of what form it may take, the available data clearly point to the need for more research that directly addresses the possibility of an important role for plant innate immunity in RNS. This aspect has been understudied for some time and sufficient evidence has now accumulated to strongly suggest that important information would come from such research. Using plant pathogen–host research as an example, one would expect that knowledge would emerge that could enhance the use of RNS in agriculture. For example: efforts to avoid inoculant competition with indigenous soil rhizobia that currently limits effectiveness or information that would increase nodulation under stressful environments or allow greater levels of biological nitrogen fixation.

## Conflict of Interest Statement

The authors declare that the research was conducted in the absence of any commercial or financial relationships that could be construed as a potential conflict of interest.
